# Short antimicrobial peptides as cosmetic ingredients to deter dermatological pathogens

**DOI:** 10.1007/s00253-015-6926-1

**Published:** 2015-08-26

**Authors:** Mohammad Rahnamaeian, Andreas Vilcinskas

**Affiliations:** Fraunhofer Institute for Molecular Biology and Applied Ecology, Department of Bioresources, Winchester Strasse 2, 35394 Giessen, Germany; Institute for Phytopathology and Applied Zoology, Justus-Liebig-University of Giessen, Heinrich-Buff-Ring 26-32, 35392 Giessen, Germany

**Keywords:** Anti-infective, Antimicrobial peptides, Cosmetic industry, Dermal pathogens, Prophylaxis, Skin diseases

## Abstract

Antimicrobial peptides (AMPs) are components of the innate immune system in many species of animals. Their diverse spectrum of activity against microbial pathogens, both as innate defense molecules and immunomodulators, makes them attractive candidates for the development of a new generation of antibiotics. Although the potential immunogenicity of AMPs means they are not suitable for injection and their susceptibility to digestive peptidases is likely to reduce their oral efficacy, they are ideal for topical formulations such as lotions, creams, shampoos, and wound dressings and could therefore be valuable products for the cosmetic industry. In this context, short AMPs (<20 amino acids) lacking disulfide bonds combine optimal antimicrobial activity with inexpensive chemical synthesis and are therefore more compatible with large-scale production and the modifications required to ensure stability, low toxicity, and microbial specificity. Proof-of-concept for the application of AMPs as novel anti-infectives has already been provided in clinical trials. This perspective considers the anti-infective properties of short AMPs lacking disulfide bonds, which are active against dermatologically important microflora. We consider the challenges that need to be addressed to facilitate the prophylactic application of AMPs in personal care products.

## Introduction

Animals are covered in protective layers of cells and their secretions, providing a semi-permeable environmental barrier colloquially described as “skin”. In humans, the skin is the first line of defense against continuous assault by a diverse range of microbes, some of which are able to cause severe dermatological diseases, especially in immunocompromised patients (Table 1). The innate immune response in most animal species includes the synthesis of antimicrobial peptides (AMPs) that can kill microbes by contact, suppress their growth, or act as immunomodulators (Bolouri Moghaddam et al. 2015; Rahnamaeian et al. 2009; Vilcinskas 2011). Hundreds of AMPs have been discovered with overlapping activity spectra against different classes of pathogen (including viruses, bacteria and fungi), and this makes them attractive candidates for the development of novel antimicrobials. Because they are peptides, they are unlikely to be suitable for injection (due to potential immunogenicity) or oral administration (due to peptidase sensitivity) without significant modification (Wiesner and Vilcinskas 2010), leaving topical application as the one remaining option. Humans naturally produce AMPs in the skin, so the addition of further AMPs with broader activity could be developed as a therapeutic intervention for the treatment of skin diseases and also a prophylactic strategy in the cosmetic industry to deter pathogens and promote skin health. This could be realized in topical formulations such as ointments, lotions, shampoos, creams, or wound dressings targeting skin pathogens and avoiding the complexities of systemic anti-infectives (Table 2).

**Table 1:** Dermatological diseases, corresponding pathogens and relevant AMPs

Disease	Microbial agent	Region of infection	Active AMPs	Reference
Folliculitis	*Staphylococcus aureus*	Hair follicles	UBI	Brouwer et al. 2006
			TsAP-2	Guo et al. 2013
			NRC-16	Gopal et al. 2013
			Epinecidin 4 & 5 & 6 & 7 & 8	Lin et al. 2013
			PE1 & PE2	Huang et al. 2013
			HM2 & HM5	Park et al. 2004
			Ranalexin	Aleinein et al. 2013
			Plc-2	Souza et al. 2013
Hot tub folliculitis	*Pseudomonas aeruginosa*	Hair follicles	Stylisin 2	Dahiya & Gautam, 2010
			NRC-16	Gopal et al. 2013
			PE1 & PE2	Huang et al. 2013
			Plc-2	Souza et al. 2013
Impetigo	*Staphylococcus aureus*	Skin	See above	See above
	*Staphylococcus pyogenes*		Ranalexin	Aleinein et al. 2013
Dermatophytosis	*Microsporum audouinii*	Skin	Stylisin 2	Dahiya & Gautam, 2010
	*Microsporum gypseum*		Tachyplesin III	Simonetti et al. 2009
White piedra	*Trichosporon beigelii*	Hair	HM2 & HM5	Huang et al. 2013
			Rev-NIS	Lee & Lee 2009
Seborrheic dermatitis	*Malassezia furfur*	Scalp, face, torso	P5	Ryu et al. 2011
			Rev-NIS	Lee & Lee 2009
Onychomycosis	*Trichophyton rubrum*	Nail	Cryptocandin	Stobel et al. 1999
			Tachyplesin III	Simonetti et al. 2009
	*Trichophyton mentagrophytes*	Nail	Stylisin 2	Dahiya & Gautam 2010
			A12-C	Gálvez et al. 1993
			Cryptocandin	Stobel et al. 1999
			Tachyplesin III	Simonetti et al. 2009
Athlete’s foot (tinea pedi)	*Trichophyton rubrum*	Foot	See above	See above
Intertrigo	*Candida albicans*	Body folds	Stylisin 2	Dahiya & Gautam 2010
			TsAP-2	Guo et al. 2013
			HM2 & HM5	Park et al. 2004
			Cryptocandin	Stobel et al. 1999
			Ranalexin	Aleinein et al. 2013
			Rev-NIS	Lee & Lee 2009
Jock itch/tinea cruris	*Trichophyton rubrum*	Groin	See above	See above
Ringworm—tinea corporis	*Trichophyton rubrum*	Glabrous skin	See above	See above
	*Microsporum canis*		A12-C	Gálvez et al. 1993
			Tachyplesin III	Simonetti et al. 2009
	*Trichophyton mentagrophytes*		See above	See above
Tinea versicolor	*Malassezia furfur*	Trunk and proximal extremities	See above	See above
Atopic eczema and seborrhoeic eczema, dandruff	*Malassezia sympodialis*	Skin	Arg9	Holm et al. 2012
			Tat (47–57)	
			Pnetratin	
			pVEC	
			Scrambled pVEC	
Tinea barbae	*Trichophyton mentagrophytes*	Hair and beard	See above	See above

**Table 2:** Properties of AMPs that are suitable for inclusion in cosmetic products

Name	Origin	aa sequence (length)	Against	MIC	Hemolytic	Reference
UBI	Ubiquitin-der	RAKRRMQY (8)	MRSA	16 (IC50), 19.5 (IC90), 29.5 (IC99) μM^a^	N.D	Brouwer et al. 2006
Stylisin 2	Synthetic	PIPFPPY (7)	*P. aeruginosa*, *C. albicans*, *Trichophyton mentagrophytes*, *Microsporum audouinii*	6–25 μg/mL	N.D.	Dahiya & Gautam, 2010
TsAP-2	*Tityus serrulatus*	FLGMIPGLIGGLISAFK(17)	*S. aureus*, *C. albicans*	5–10 μM	18 % at 20 μM	Guo et al. 2013
NRC-16	Witch-flounder	GWKKWLRKGAKHLGQAAIK (19)	*P. aeruginosa*, *S. aureus* ^b^	1–8 μM	No	Gopal et al. 2013
Epinecidin-4	Grouper (fish)	FIFHIIKGLFH (11)	*S. aureus*	6.25 μg/mL	Not up to 12.5 μg/mL	Lin e al. 2013
Epinecidin-5		FIFHIIKGLF (10)				
Epinecidin-6		FIFHIIKGLFHA (12)				
Epinecidin-7		FIFHIIKGLFHAG (13)				
Epinecidin-8		FIFHIIKGLFHAGKMI (16)				
PE1/PE2	*Paenobacillus ehimensis* B7	I/LDab^c^FL/IDab^c^VL/IT (8)	Pan-drug-resistant *P. aeruginosa*, *MRSA*	2–8 μg/mL	N.D.	Huang et al. 2013
HM2	Synthetic	AKKVFKRLGIGAVLKVLTWG (20)	*S. aureus*	0.78 μM	No	Park et al. 2004
				25–50 μM		
			*Trichosporon begelii*	50 μM		
			*C. albicans*			
HM5	Synthetic	AKKVFKRLGIGAVLKVLKKG (20)	*S. aureus*	0.78 μM	No	Park et al. 2004
			*T. begelii*	3.125–6.25 μM		
			*C. albicans*	6.25 μM		
P5: Cecropin (1–8)-Magainin2 (1–12)	Synthetic	KWKKLLKKPLLKKLLKKL-NH_2_ (18)	*M. furfur.*	0.39 μM	N.D.^e^	Ryu et al. 2011
A12-C	*Bacillus licheniformis* A12	QQRAPYOrn^d^ (7)	*Microsporum canis*, *Trichophyton mentagrophytes*	470 AU/mg protein	N.D.	Gálvez et al. 1993
				3120 AU/mg protein		
Cryptocandin	*Cryptosporiopsis quercina*	QPOrn^d^TPhT^e^ (6)	*T. mentagrophytes*, *T. rubrum*, *C. albicans*	0.03–0.07 μg/mL^f^	N.D.	Stobel et al. 1999
(rocombinant) Ranalexin	*Rana catesbiana*	FLGGLIKIVPAMICAVTKKC (20)	*S. aureus*, *S. pyogensis*, *MRSA*, *C. albicans*	8 μg/mL	Cytotoxic to cancer cells^g^	Aleinein et al. 2013
Plc-2	Pleurocidin	KHVGKAALTHYL (12)	*S. auresu*	4.6 μM	Yes^h^	Souza et al. 2013
			*P. aeruginosa*	9.1 μM		
Arg 9	Synthetic	RRRRRRRRR (9)	*Malassezia sympodialis*	14 μg/mL	N.D.^i^	Holm et al. 2012
Tat (47–57)	Synthetic	YGRKKRRQRRR (11)	*M. sympodialis*	>17.2 μg/mL	N.D.^i^	
Penetratin	Synthetic	RQIKIWFQNRRMKWKK (16)	*M. sympodialis*	>22.5 μg/mL	N.D.^i^	
pVEC	Synthetic	LLIILRRRIRKQAHAHSK (18)	*M. sympodialis*	11 μg/mL	N.D.^i^	
Scrambled pVEC	Synthetic	IAARIKLRSRQHIKLRHL (18)	*M. sympodialis*	>22.1 μg/mL	N.D.^i^	
Rev-NIS (nuclear entry inhibitory signal peptide of Rev. protein)	HIV-1 Rev. protein	ELLKAVRLIK (10)	*C. albicans*	10 μM	N.D.^i^	Lee and Lee 2009
			*M. furfur.*	20 μM		
			*T. beigelii*	20–40 μM		

*ND* ^n^Not determined

^a^Data are expressed as inhibition concentration (IC) to eradicate 50 % (IC 50), 90 % (IC 90), or 99 % (IC 99) of the number of viable microorganisms as compared to control incubations

^b^Resistant strains of *P. aeruginosa* isolated from patients with otitis media in a hospital, including Flomoxef sodium-, Cefrpiramide-, and Isepamicin-resistant *P. aeruginosa*; resistant strains of *S. aureus* isolated from patients in a hospital

^c^2,4-Diaminobutyric acid

^d^Orn: Ornithine

^e^ hT: homoTyrosine

^f^ Plate MIC

^g^Cytotoxic to HeLa and COS7 cells with IC50 values of 13.4 and 14.8 μg/mL, respectively

^h^Much lower hemolytic effect compared to melittin and other well-known antibiotics, such as ampicillin, vancomycin, cefotaxime, chloramphenicol and kanamycin

^i^Non-cytotoxic effect on human keratinocytes

The production of AMPs as cosmetic ingredients would require large-scale production, placing the focus on properties such as peptide length and structure. AMPs longer than 20 amino acids would be too expensive for routine chemical synthesis, and the requirement for disulfide bonds would introduce additional folding steps that would also increase the costs of production. Short AMPs are less immunogenic and would therefore be less likely to trigger allergies (Dhiraj et al. 2006), and they would be easier to modify to increase stability, reduce toxicity, or engineer microbial specificity. It is worthy to note that the selection of an appropriate formulation for AMP-based topical products has a substantial impact on their efficacy (Trotti et al. 2004; Kollef et al. 2006; van Saene et al. 2007) as shown for the treatment of oral mucositis, in which a mucosal-adhesive paste is superior to a mouthwash (van Saene et al. 2007).

In this perspective, we discuss the antimicrobial properties of short AMPs (fewer than 20 residues, and no disulfide bonds) that are active against dermatologically relevant pathogens as cosmetic ingredients. We discuss the requirements that must be met for their prophylactic application in personal care products. However, we do not include the development of AMPs targeting *Acne vulgaris*, which has been comprehensively discussed in other articles (Fisk et al. 2014; Howard 2014; Cresce et al. 2014; Bartlett et al. 2014; Rocha et al. 2014; Zouboulis 2014).

## Naturally occurring AMPs in human skin

Like other animals, humans naturally produce AMPs in the skin to deter pathogens. These self-peptides are suitable candidates for development as cosmetic ingredients because they would be the least likely to cause off-target effects. Human skin AMPs include β-defensin (hBD-2), RNase7, and psoriasin, which are active against different ranges of skin pathogens. The antimicrobial activities of these peptides have been compared to the antibiotic fluconazole against the germinating conidia of *Trichophyton rubrum*, *Trichophyton mentagrophytes*, *Epidermophyton floccosum*, and *Microsporum canis*, revealing that all three AMPs significantly inhibited fungal growth although with variable efficacy depending on the AMP/pathogen combination. *E. floccosum* was suppressed by all three AMPs, whereas *M. canis* was only inhibited by psoriasin, but in the latter case, the inhibitory effect was greater than fluconazole (Fritz et al. 2012). Unfortunately, all three of the AMPs discussed above exceed 20 amino acids in length or contain disulfide bonds, which would increase the complexity and expense of large-scale production.

## AMP-derived core fragments and their activity against skin pathogens

Although natural human AMPs may be too long or complex for chemical synthesis, the entire AMP might not be required because the antimicrobial activity resolves to a smaller essential motif. This shorter derivative might show the same efficacy as the parent peptide but would be easier to synthesize. Several reports describe the engineering of AMPs to retain or even improve their efficacy while reducing their length as much as possible. One example is UBI, an eight-residue synthetic peptide representing residues 31–38 of the 59-residue full-length AMP ubiquicidin, which retains full activity against methicillin resistant *Staphylococcus aureus* (Brouwer et al. 2006). Likewise, Lin et al. (2013) synthesized a series of truncated AMPs (anti-lipopolysaccharide factor from shrimp, epinecidin from grouper, and pardaxin from *Pardachirus marmoratus*) to look for shorter stretches of sequences that increase antimicrobial potency. As a result, epinecidin-8 and pardaxin-6 showed a broad range of activity against both Gram-positive and Gram-negative bacteria, with no induction of hemolysis. The same is true for Plc-2 which is a 12-residue C-terminal fragment of pleurocidin and the smallest fragment that retains the antimicrobial potency of the original peptide. Based on MIC values determined in vitro with low-ionic-strength medium, Plc-2 was active against *S. aureus*, *Escherichia coli*, and *Pseudomonas aeruginosa* but not against *Enterococcus faecalis*. The antifungal activity of the synthetic peptides against plant pathogens such as *Fusarium oxysporum* and *Colletotrichum* sp. were shown to be mediated by the lysis of organelle membranes (Souza et al. 2013).

## Engineering of AMPs to maximize antimicrobial activity and minimize hemolytic effect

One concern regarding AMPs is their possible hemolytic effects at higher concentrations, which necessitates their targeted engineering to (i) improve their antimicrobial activity and (ii) reduce their hemolytic effects. Generally, an increase in positive charge increases the antimicrobial activity whereas an increase in hydrophobicity causes a hemolytic effect (Park et al. 2004). The configurational stereochemistry (D or L enantiomers) of specific amino acids may also affect hemolytic activity (Roy et al. 2002). In an attempt to trim AMPs by Lys-substitution to improve their net charge, Park et al. (2004) produced the peptides HM2 and HM5, which showed antimicrobial activity against *S. aureus*, *Trichospora beigelii* and *Candida albicans* but no hemolytic activity in the range of 0.78–50 μM. In another study, Guo et al. (2013) tested two 17-mer amidated linear peptides (TsAP-1 and TsAP-2) isolated from the Brazilian yellow scorpion (*Tityus serrulatus*) and found they have distinct antimicrobial properties. TsAP-1 was less active than TsAP-2 against *E. coli*, *S. aureus*, and *C. albicans* with minimal inhibitory concentrations (MICs) of 120–160 μM and hemolytic activity of 6.48 % at 160 μM, whereas TsAP-2 showed potent activity against *S. aureus* (MIC =5 μM) and *C. albicans* (MIC = 10 μM) with almost no hemolytic activity at ≤15 μM but strong hemolytic activity thereafter. The replacement of four neutral amino acid residues in each peptide with lysine residues had a dramatic effect on their antimicrobial and hemolytic activities, particularly those of TsAP-1. The MIC values for the enhanced cationic analog (TsAP-S1) fell to 2.5 μM for *S. aureus* and *C. albicans* and 5 μM for *E. coli*, but the hemolytic activity increased to a maximum of 28 % at 5 μM. The same lysine residue substitutions in TsAP-2 reduced its MIC against *E. coli* from >320 to 5 μM, but the hemolytic activity remained as high as TsAP-S1 (Guo et al. 2013).

## AMPs promising for the skin infections and atopic eczema

*C. albicans* can cause skin infections and atopic eczema, characterized by the accumulation of neutrophils culminating in inflammatory responses and the secretion of enzymatic proteins. High-molecular-weight kininogen (HK) then triggers the formation and release of AMPs to interfere with the establishment of pathogenesis. Highly potent antifungal peptide fragments are produced via the proteolytic degradation of HK (Sonesson et al. 2011). Lipophilic yeasts of the genus *Malassezia* cause seborrheic dermatitis, resulting in the colonization of infected skin by *Malassezia furfur*, *Malassezia globosa*, *Malassezia restricta*, *Malassezia slooffiae*, *Malassezia sympodialis*, or *Malassezia obobtusa* (Nakabayashi et al. 2000). Especially, *M. furfur* causes several recalcitrant skin infections such as *Tinia versicolor*, seborrheic dermatitis and folliculitis. Pro-inflammatory cytokines produced at infected sites by keratinocytes then induce the inflammatory response, and saturated fatty acids released by yeast lipases stimulate the proliferation of yeast cells (Bukvić Mokos et al. 2012). Current therapeutic approaches involve topical formulations containing antifungal and anti-inflammatory agents, except severe and recalcitrant cases which require systemic intervention. In the majority of cases, the first choice treatment is topical azole-type antifungal agents.

Recalcitrant skin infections caused by *M. furfur* are commonly treated with ketoconazole and/or itraconazole. However, certain AMPs are active against *M. furfur*, including cathelicidin (López-García et al. 2006). The infection of human keratinocytes with *M. furfur* also upregulates endogenous hBD-2 (Donnarumma et al. 2004). The antifungal and anti-inflammatory effects of cecropin A(1-8)-magainin 2(1-12) hybrid peptide analog P5 against *M. furfur* were reported by Ryu et al. (2011). The MIC of P5 against *M. furfur* was 0.39 μM, 3–4 times higher than ketoconazole and itraconazole. From the mechanistic point of view, expression of IL-8 and Toll-like receptor 2, the activation of NF-κB, and the release of intracellular calcium in *M. furfur*-infected human keratinocytes were strongly attenuated by treatment with P5. Rev-NIS (nuclear entry inhibitory signal peptide of Rev protein), which is a 10-residue derivative of HIV-1 Rev protein also acts against *M. furfur* with MIC value of 20 μM (Lee and Lee 2009).

## Microorganisms-derived AMPs active against dermal pathogens

Fungal AMPs also show promising antimicrobial activity. The acidic hydrophilic heat-stable 0.77 kD peptide A12-C from *Bacillus licheniformis* (Glu-Glu-Arg-Ala-Pro-Tyr-Orn) has been shown to inhibit cell growth and hyphal proliferation in *Mucor plumbeus*, *Mucor mucedo*, and the human dermal pathogens *M. canis* and *T. mentagrophytes* (Gálvez et al. 1993). A12-C is resistant to the proteolytic enzymes pronase, trypsin, and proteinase K. As well, cryptocandin A is an aromatic lipopeptide antifungal agent isolated from the endophytic fungus *Cryptosporiopsis* cf. *quercina*, which shows antifungal activity against *T. mentagrophytes* and *T. rubrum* with plate MIC values of 0.035–0.070 μg ml^−1^ (Strobel et al. 1999). A plate MIC value of 0.035 μg ml^−1^ was recorded against *C. albicans*, making this peptide and its chemical derivatives echinocandin and pneumocandin promising candidate therapeutics particularly for the control of human nail and skin diseases such onychomycosis, tinea pedi, tinea cruris, tinea corporis, and tinea barbea.

Recently, Huang et al. (2013) reported two active compounds (PE1 and PE2) isolated from *Paenibacillus ehimensis* B7, which are active against clinical isolates of *E. coli*, pan-drug-resistant *P. aeruginosa*, and methicillin-resistant *S. aureus*.

## Insect-derived AMPs

Insects represent the most diverse group of organism on earth. Their biodiversity at the species level is also reflected by their diversity of AMPs. The evolutionary success of insects can at least in part be attributed to their innate immune systems which display a remarkable plasticity in terms of functional shifts, diversification, or loss of genes encoding AMPs (Vilcinskas 2013). The highest number of AMPs in a single organism, which is more than 50 different AMPs, has been found in the invasive ladybird *Harmonia axyridis* (Vilcinskas et al. 2013). Insect-derived AMPs have been suggested for the development of novel anti-infectives (Ratcliffe et al. 2011; Vilcinskas 2013; Yi et al. 2014). Of particular interest for the development of directly applicable peptides against skin pathogens are AMPs from medicinal maggots of the green bottle fly *Lucilia sericata.* Their secretions cause remarkable therapeutic effects even on chronic and nonhealing wounds which include the removal of necrotic tissue (debridement), the acceleration of wound healing and wound disinfection (Sherman 2014). The value of medicinal maggots which are also known as wound maggots in traditional medicine has inspired researchers to identify AMPs produced and secreted by the maggots. A defensin-like peptide named lucifensin displays activity against Gram-positive bacteria (Cerovsky et al. 2010). Another novel peptide from *L. sericata* which displays exclusive antifungal activities has been named lucimycin (Pöppel et al. 2014). However, both AMPs are too large to be developed as short peptidic anti-infectives. Experimental screening for immune-inducible AMPs using suppression subtractive hybridization (Altincicek and Vilcinskas 2009) and next-generation sequencing of the transcriptome from immune-challenged *L. sericata* maggots identified 47 genes encoding putative AMPs including short peptides such as cecropins, diptericins, proline-rich peptides, and sarcotoxins. Twenty three of them were produced as synthetic analogs among which some exhibited activity against a broad spectrum of microbial pathogens (Pöppel et al. 2015). The easy-to-synthesize *Lucilia*-derived AMPs with potent antimicrobial activity are presently produced in bulk to enable their development both as constituents in hydrogels for wound healing and as cosmetic ingredients to deter dermatological pathogens.

## Skin pathogens can modulate the efficacy of AMPs

*C. albicans* secretes a large glycofragment of the Msb2 surface protein (Msb2*) into its immediate environment as a protective decoy for the human AMPs LL-37 and histatin-5 (Swidergall et al. 2013). Likewise, Msb2* can also interact with human defensins thereby shielding *C. albicans* from their antifungal properties. It also inactivates the lipopeptide antibiotic daptomycin, protecting *S. aureus*, *Corynebacterium pseudodiphteriticum*, and *E. faecalis*. The concurrent cultivation of *S. aureus* and wild-type *C. albicans* (but not the *msb2* mutant) resulted in the cross-protection of the bacteria by Msb2* which attenuated the bactericidal activity of daptomycin and thus incapacitated an important reserve antibiotic (Swidergall et al. 2013).

Cationic AMPs often work by penetrating the bacterial cell membrane and altering its transmembrane potential (Rahnamaeian 2011). Accordingly, sense–response systems that perceive AMPs and induce adaptive reactions can increase bacterial persistence. In *S. aureus*, the LytSR two-component regulatory system may serve as a sensor of transmembrane potential. According to Yang et al. (2013), LytSR plays a key role in the adaptive response of *S. aureus* to membrane-stressing AMPs, perhaps by perceiving slight changes in transmembrane potential.

## Cationic AMPs are sensitive to pH

Walkenhorst et al. (2013) described a family of cationic AMPs with strong, broad-spectrum activity at neutral pH and low ionic strength. The efficacy of these peptides against an array of microbes varied linearly with pH for each subtype, i.e. they were far less effective against *C. albicans* and Gram-negative bacteria at high pH values, reflecting the decline in net positive charge. Interestingly, a reverse pH trend was observed for their activity against the Gram-positive bacterium *S. aureus*, probably reflecting the presence of charged molecules in the peptidoglycan layer of the cell wall. The buffer pH and ionic strength showed an additive effect on AMP activity (Walkenhorst et al. 2013).

## Multiple AMPs can interact to maximize their antimicrobial potency

The synergy of AMPs and antibiotics has been frequently reported. For instance, Ranalexin is a cationic peptide 20 residues in length isolated from *Rana catesbeiana*, which is active against *S. aureus*, *S. pyogenesis*, methicillin-resistant *S. aureus*, and *C. albicans* with a MIC value of 8 μg ml^−1^ (Aleinein et al. 2013); however, when combined with polymyxin B or linezolid, the MIC value is reduced by 75 % (Aleinein et al. 2014). As well, epinecidin-8 and pardaxin-6 work in synergy with streptomycin and kanamycin to increase their potency up to 75 % against methicillin-resistant *S. aureus* (Lin et al. 2013). Nonetheless, the potentiation between naturally co-occurring AMPs has been almost neglected. We have recently shown that the concurrent application of multiple AMPs can increase the overall antimicrobial potency in a more than additive manner (Rahnamaeian et al. 2015; Pöppel et al. 2015). In nature, microbial challenge elicits a cocktail of AMPs in response (Rahnamaeian and Vilcinskas 2012; Vilcinskas et al. 2013). These AMPs usually have diverse modes of action so that they complement rather than replicate each other. For example, the insect AMPs abaecin and hymenoptaecin work cooperatively to inhibit the proliferation of *E. coli* at low MICs (Rahnamaeian et al. 2015). Abaecin shows no antimicrobial activity when presented alone because it needs to gain entry into the cell to interact with the bacterial chaperone DnaK, whereas hymenoptaecin is only active at high concentrations because its mechanism as a sole agent is based on the dose-dependent perforation of the bacterial membrane. However, when both AMPs are present together, hymenoptaecin creates pores that allow abaecin to access the cell and bind to DnaK, and this in turn inhibits the bacterial mechanisms that repair membrane damage allowing hymenoptaecin to compromise the cell more rapidly. A similar synergistic activity has also been observed among particular AMPs produced and secreted by medicinal maggots *L. sericata* (Pöppel et al. 2015). Because the antimicrobial properties of AMPs can be resolved to shorter motifs, the synthesis of hybrid peptides encompassing the functional motifs of several AMPs could provide robust protection against microbial pathogens by combining the beneficial properties of multiple AMPs into one therapeutic entity.

## Organometallic derivatives of short AMPs

**Fig. 1:**
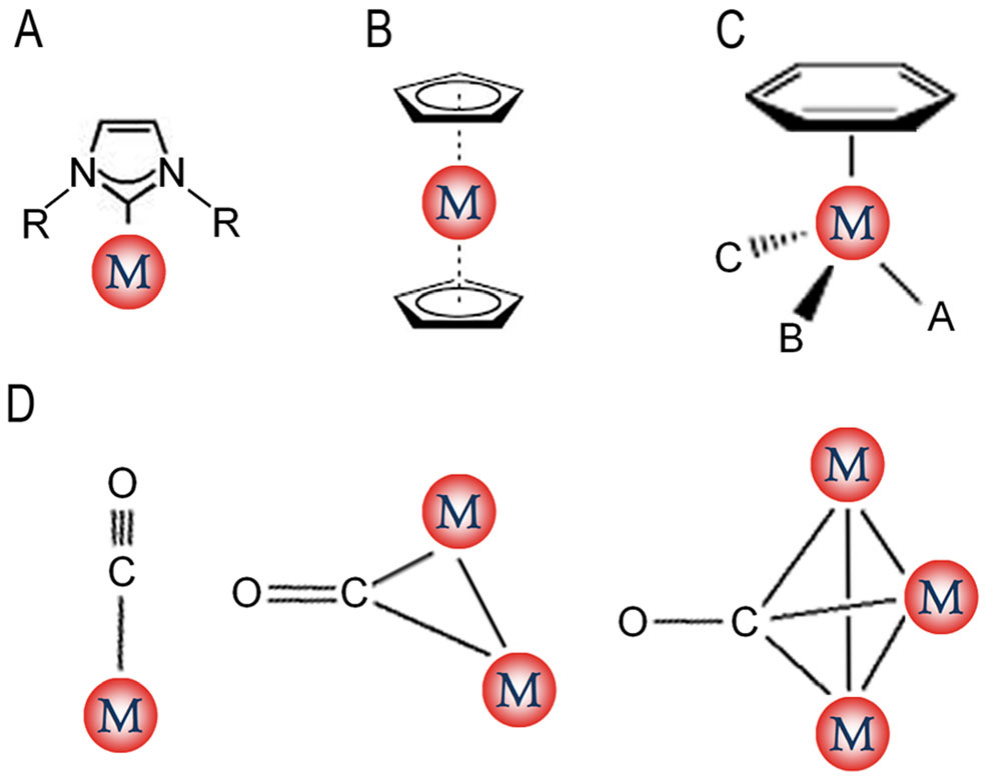
Classic groups of the organometallics used in medicinal chemistry. **a** Metal-carbene. **b** Metallocene. **c** Metal-arene. **d** Metal-carbonyl (in different variants). *M* indicates the metal element

The organometallic derivation of existing short AMPs has the potential to (i) increase the potency of AMPs and (ii) provide metal-specific modes of action that delay the acquisition of resistance by bacterial populations. The typical classes of organometallic compounds are metallocene, M-arene, M-carbonyl, and M-carbene (Fig. 1). The activity of ferrocenoyl-derivatized AMPs predominantly reflects the lipophilic properties of the organometallic fragment (Albada et al. 2013). The L-to-D substitution of amino acid residues in short lipidated AMPs can also produce diastereomeric peptides with optimized antimicrobial potency and reduced hemolytic activity (Albada et al. 2013). The structural diversity and potential catalytic properties of organometallic AMP derivatives and their availability for ligand exchange and redox reactions (Gasser and Metzler-Nolte 2012) may contribute to their development as cosmetic ingredients. Generally, transition metals of the second and third rows of the periodic table with closed electron shells, e.g., ruthenium (Ru), osmium (Os), and iridium (Ir), are the most proper cases to be recruited in medicinal inorganic chemistry (Gasser and Metzler-Nolte 2012).

## Conclusion

Although AMPs have only been tested in a small number of clinical trials, there is adequate proof-of-concept to support their use as topical anti-infectives. Antimicrobials and their derivatives are unlikely to replace conventional antibiotics because they cannot be administered orally or by injection, but they are ideal for topical applications and could be used as pharmaceuticals to treat skin infections as well as cosmetic ingredients to deter skin pathogens and maintain skin health. The diverse spectrum of antimicrobial activities demonstrated by different AMPs, and the ease with which short AMPs can be synthesized and modified, make them promising candidates for the development of cosmetic products that deter dermatological pathogens.
